# Endothelial to Mesenchymal Transition in Pulmonary Vascular Diseases

**DOI:** 10.3390/biomedicines8120639

**Published:** 2020-12-21

**Authors:** Eunsik Yun, Yunjin Kook, Kyung Hyun Yoo, Keun Il Kim, Myeong-Sok Lee, Jongmin Kim, Aram Lee

**Affiliations:** 1 Division of Biological Sciences, Sookmyung Women’s University, Seoul 04310, Korea; yes951212@naver.com (E.Y.); jenny1267@naver.com (Y.K.); khryu@sookmyung.ac.kr (K.H.Y.); kikim@sookmyung.ac.kr (K.I.K.); mslee@sookmyung.ac.kr (M.-S.L.); 2 Research Institute for Women’s Health, Sookmyung Women’s University, Seoul 04310, Korea

**Keywords:** lung disease, endothelial to mesenchymal transition, pulmonary hypertension, pulmonary fibrosis

## Abstract

Lung diseases, such as pulmonary hypertension and pulmonary fibrosis, are life-threatening diseases and have common features of vascular remodeling. During progression, extracellular matrix protein deposition and dysregulation of proteolytic enzymes occurs, which results in vascular stiffness and dysfunction. Although vasodilators or anti-fibrotic therapy have been mainly used as therapy owing to these characteristics, their effectiveness does not meet expectations. Therefore, a better understanding of the etiology and new therapeutic approaches are needed. Endothelial cells (ECs) line the inner walls of blood vessels and maintain vascular homeostasis by protecting vascular cells from pathological stimuli. Chronic stimulation of ECs by various factors, including pro-inflammatory cytokines and hypoxia, leads to ECs undergoing an imbalance of endothelial homeostasis, which results in endothelial dysfunction and is closely associated with vascular diseases. Emerging studies suggest that endothelial to mesenchymal transition (EndMT) contributes to endothelial dysfunction and plays a key role in the pathogenesis of vascular diseases. EndMT is a process by which ECs lose their markers and show mesenchymal-like morphological changes, and gain mesenchymal cell markers. Despite the efforts to elucidate these molecular mechanisms, the role of EndMT in the pathogenesis of lung disease still requires further investigation. Here, we review the importance of EndMT in the pathogenesis of pulmonary vascular diseases and discuss various signaling pathways and mediators involved in the EndMT process. Furthermore, we will provide insight into the therapeutic potential of targeting EndMT.

## 1. Introduction

**Figure 1:**
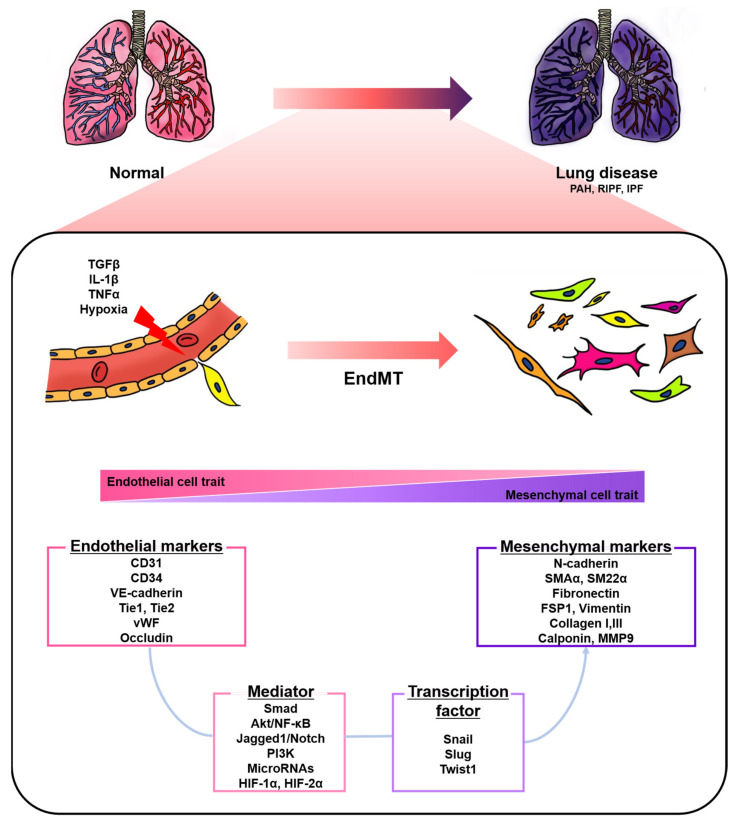
A schematic representation of endothelial-to-mesenchymal transition (EndMT) involved in lung diseases. Endothelial cells stimulated by transforming growth factor-β (TGFβ), interleukin 1 beta (IL-1β), tumor necrosis factor alpha (TNFα), and hypoxia undergo EndMT. EndMT is characterized by phenotypic change from a cobblestone into an elongated shape, loss of endothelial markers, and the acquisition of mesenchymal markers. EndMT contributes to the pathogenesis of lung diseases, including pulmonary arterial hypertension (PAH), radiation-induced pulmonary fibrosis (RIPF), and idiopathic pulmonary fibrosis (IPF). Various mediators and transcription factors are identified in this process.

Endothelial cells (ECs), a monolayer composed of the inner cellular lining of the vascular lumen, play an important role in various physiological processes to maintain vascular homeostasis [1,2,3]. These cells are involved in the regulation of vascular tone, permeability, and inflammatory responses [4]. However, endothelial injury by stimuli, such as hypoxia, pro-inflammatory cytokines and abnormal mechanical forces, can induce endothelial-to-mesenchymal transition (EndMT), resulting in endothelial dysfunction and destruction of homeostasis [2,5]. EndMT is the process by which ECs lose their cellular features and acquire mesenchymal characteristics [6]. EndMT-derived cells gain migration potential by losing endothelial markers, such as cluster of differentiation 31 (CD31) and vascular endothelial cadherin (VE-cadherin), which are involved in cell-to-cell contact [7,8]. Concomitantly, the expressions of mesenchymal markers, such as fibronectin, alpha-smooth muscle actin (SMAα), smooth muscle protein 22 alpha, vimentin, and neural cadherin (N-cadherin), are upregulated [7,8]. The morphology of ECs undergoing EndMT changes from a cobblestone monolayer to an elongated phenotype [9]. This phenomenon mainly occurs during embryonic cardiac development, but is also involved in various lung diseases, such as pulmonary arterial hypertension (PAH) and pulmonary fibrosis (PF) (Figure 1) [7,10,11,12,13].

Pulmonary hypertension (PH) is categorized into five groups: PAH, PH due to left heart disease, PH due to lung diseases and/or hypoxia, PH due to pulmonary arterial obstructions, and PH with unclear and/or multifactorial mechanisms [14,15,16]. PAH has been defined as pulmonary artery pressure (PAP) ≥ 25 mmHg at rest and occurs as a result of multiple causes, such as heritable factors (mainly bone morphogenic protein receptor-2 (BMPR2) mutations), drugs and toxins, as well as association with other diseases; however, PAH without known causes is known as idiopathic PAH (IPAH) [14,17]. Vascular remodeling in PAH is characterized by the aberrant proliferation of pulmonary arterial ECs (PAECs) and smooth muscle cells (SMCs), which form occlusive neointima and vascular structural changes [18,19,20]. These progressive changes cause excess vasoconstriction and right ventricle hypertrophy and, ultimately, death [18,19,20]. Endothelial dysfunction is a key player in the pathogenesis of PAH [21]. Growing evidence suggests that EndMT potentially contributes to endothelial dysfunction and the vascular remodeling of PAH [7,11,22,23]. Indeed, many studies have demonstrated that various signaling pathways and mediators, including transforming growth factor beta (TGFβ), nuclear factor kappa B (NF-κB), Notch, and microRNA, are involved in the EndMT of PAH [24,25]. It has been reported that the endothelial-specific loss of BMPR2, known as the principal mutation factor of heritable PAH, induces EndMT in vitro and in vivo [7,11,23]. In addition, exposure to hypoxia or chronic stimulation with proinflammatory cytokines or TGFβ also induce EndMT in vitro and in vivo [26,27,28,29,30]. However, the contribution of EndMT to disease progression is not fully understood [2]. Current therapies for PAH, such as phosphodiesterase-5 inhibitors, prostacyclin analogues, and endothelin receptor antagonists, can help relieve symptoms and slow progression, but there is no effective treatment [21,31]. Thus, targeting EndMT is emerging as a novel therapeutic approach by alleviating vascular remodeling and the PAH phenotype in vitro and in vivo [7,29,32,33,34,35,36].

Idiopathic PF (IPF) is chronic, progressive, and the most common interstitial lung disease without a definite etiology [37,38]. Various cell types, such as epithelial cells, pneumocytes, ECs, pericytes, fibrocytes, resident fibroblasts, and mesenchymal cells, are associated with the pathogenesis of IPF [25]. The injured epithelial cells, through aging, genetic susceptibility and repetitive microinjury, release fibrogenic factors and cytokines, resulting in the recruitment of contractile myofibroblasts, which are key cellular mediators of fibrosis [38]. Recruited myofibroblasts undergoing activation and proliferation induce extracellular matrix expansion, which consequently results in aberrant vascular remodeling in the lung [38]. The myofibroblasts are derived not only from the proliferation of resident mesenchymal cells, circulating fibrocytes, lung interstitium pericytes, epithelial–mesenchymal transition, but also EndMT. [38,39,40]. Many studies have demonstrated that EndMT occurs in the lung tissue of IPF patients and animal models, suggesting EndMT may play an important role in pathological processes in PF [25,41,42]. In addition, emerging evidence indicates that inhibiting EndMT can also be a therapeutic strategy in PF in vivo [41,43,44,45].

This review highlights the role of EndMT associated with pulmonary diseases, such as PAH and PF. Moreover, this review discusses molecular mechanisms, epigenetic modulation, and recent clinical relevance in lung diseases.

## 2. EndMT in Pulmonary Hypertension

PH is characterized by the muscularization of arterioles, medial thickening, plexiform region formation, intimal fibrosis, and the hyperproliferation of ECs and SMCs [15,16,46,47]. Most studies have identified EndMT by analyzing the co-expression of endothelial markers and mesenchymal markers in the lung tissue of patients and experimental PH animal models. EndMT has been observed in pathological lesions in the lungs of PH patients [7,30,32,48,49]. Endothelial (CD31, CD34, and VE-cadherin) and mesenchymal marker (SMAα) double-positive cells were observed in intimal and plexiform lesions in the lung tissue of PAH patients [7]. Another group also demonstrated that neointimal and plexiform lesions in the lung tissue of human PAH patients contain endothelial markers, CD31 or von Willebrand factor (vWF), and SMAα co-expressing cells [48]. Isobe et al. reported that the CD44 spliced variant form (CD44v) results from EndMT, and its positive cells also expressed vWF and SMAα in neointimal lesions of IPAH patients [32]. The 4 ± 1% of pulmonary arterioles in systemic sclerosis (SSc)-PAH patients showed vWF/SMAα co-localization [30]. CD31 and SMAα co-expressing cells were detected in endarterectomized tissues from patients with chronic thromboembolic pulmonary hypertension (CTEPH) [49].

In addition to performing the double staining of endothelial and mesenchymal markers, ECs isolated from the lung have also been used for studying EndMT [49,50]. Endothelial-like cells isolated from the vascular tissue of patients with CTEPH underwent disruption of the endothelial monolayer and abnormal growth even after sorting with CD31 [49]. In addition, conditioned media from myofibroblast-like cells isolated from CTEPH patients induced phenotypic changes and mesenchymal marker expression in pulmonary microvascular ECs (PMVECs) [49]. Pulmonary vascular ECs (PVECs) isolated from patients with IPAH exhibited molecular characteristics of EndMT and a spindle-shaped morphology, which was similar to that of normal PVECs treated with TGFβ1, a well-known factor of EndMT [50]. Pulmonary arteries isolated from PAH patients showed increased mRNA levels of mesenchymal markers and EndMT-related factors, which also supports EndMT [7].

Animal models have also been used to demonstrate EndMT. Monocrotaline (MCT) injection causes endothelial injury and pulmonary vascular remodeling, and is commonly used to induce severe PH [50,51]. Several groups observed the reduction of endothelial markers and the induction of mesenchymal markers, as well as the co-staining of SMAα and endothelial marker (CD31 or CD34), in the lung tissue of MCT-induced PH rats [7,28,29,50,52]. Zhang et al. found that changes in endothelial and mesenchymal cell marker expressions occurred in a time-dependent manner during MCT-induced PAH development [51]. Chronic hypoxia also contributes to the vascular remodeling of small pulmonary arteries [27,53]. With this, it has been demonstrated that three weeks of hypoxia induces EndMT in the pulmonary arteries of rats and mice [26,53]. EndMT was further identified within the intimal layer of small pulmonary arteries, but not in large arteries, in chronic hypoxia-induced PH rats [27]. The combination of SU5416, a vascular endothelial growth factor receptor antagonist, and a chronic hypoxia model (SuHx) has been used for severe PH owing to the similarity of pathological lesions to plexiform lesions of human PAH [53]. In the lung of the SuHx model that had over 80 mmHg of right ventricular systolic pressure (RVSP), transitions of vWF+ vimentin− ECs to vWF− vimentin high mesenchymal-like cells were observed in pulmonary vascular lesions [7]. Tie2+ vimentin+ and Tie2+ SMAα+ cells were also found in occlusive lesions [7]. In addition, 6 ± 1% of pulmonary vessels had vWF/SMAα double-positive ECs, which contrasts with normal tissues having only 1% transitional EndMT cells in SuHx mice [30].

In general, endothelial and mesenchymal marker double-positive cells are considered EndMT-induced cells. However, this approach has the limitation of not being able to distinguish complete EndMT (cEndMT), where there are lost endothelial markers, and partial EndMT (pEndMT) cells. To overcome this problem, several studies have used endothelial-specific fluorescence transgenic animals [48,54]. Qiao et al. established VE-cadherin Cre or Tie2 Cre-mTomato/mGFP lineage-tracing mice [48]. Histological analysis identified SMAα-expressing neointima in an experimental PH animal model derived from the endothelium in VE-cadherin Cre or Tie2 Cre-mTomato/mGFP lineage-tracing mice [48]. Furthermore, cEndMT cells isolated from SuHx-induced Cdh5-Cre/CAG-GFP double-transgenic mice showed a spindle-like morphology and were characterized by mesenchymal-like functions, such as high proliferation and migration ability [54]. Additionally, conditioned media from cEndMT had a paracrine effect on the proliferation and migration of non-endothelial mesenchymal cells, suggesting that EndMT contributes directly and indirectly to the vascular remodeling of PAH [54].

## 3. EndMT in Pulmonary Fibrosis

IPF characterizes matrix deposition and fibrotic tissue remodeling, and it has been demonstrated that fibroblasts are involved in pathogenesis; thus, efforts to identify the origin of fibroblasts have been made. [42,55]. In the lung tissue of radiation-induced pulmonary fibrosis (RIPF) patients and radiation-exposed mouse models, the co-localization of CD31 and SMAα was significantly elevated compared to that of the control group, indicating EndMT [41]. The same group also reported endothelial heat shock protein beta 1 (HSPB1)-dependent EndMT in the PF of lung cancer [45]. The bleomycin-induced PF in animal models is the most commonly used model to study human IPF by causing damage to epithelial cells and alveolar inflammation [56,57]. Another group reported significant alterations of EC markers in the lungs of bleomycin-treated endothelial-specific autophagy-related 7 (ATG7) knockout mice compared to bleomycin-treated WT mice [58]. Hashimoto et al. established a Tie2-Cre/CAG-CAT-LacZ double transgenic mice model to track endothelial-derived fibroblasts in bleomycin-induced PF [42]. The 16.2% of lung fibroblasts isolated from bleomycin-treated mice were X-gal-staining-positive and 14.8% of X-gal-positive cells were SMAα- and Collagen I-double positive (myofiboblast), while the other 85.2% were SMAα-negative and Collagen I-positive, suggesting that a significant number of fibroblasts are EC-derived [42]. Suzuki et al. demonstrated that PVECs isolated from lipopolysaccharide (LPS)-induced mouse lungs undergo EndMT using the double staining of CD31 and SMAα or S100A4 [59]. Flow cytometry analysis showed that the number of SMAα + PVECs and S100A4 + PVECs increased, while the total number of PVECs decreased [59].

Taken together, EndMT may play a key role in the pathogenesis of lung diseases. Many studies describe EndMT based on the evidence of co-expression of EC markers and mesenchymal markers in the lung tissue of animal disease models or human patients, which has a primary limitation because EndMT is a switching process; thus, the underlying molecular mechanisms are not yet fully understood. The methods to clarify partial and complete EndMT processes have been improved using endothelial-specific fluorescence transgenic mice; however, further investigation with human samples is needed. Thus, the clinical relevance of EndMT should be thoroughly assessed.

## 4. Key Signaling Pathways and Mediators during EndMT in Lung Diseases

The understanding of the key molecular mechanisms and mediators during EndMT is an important step toward finding how to develop EndMT inhibitors that can be applied to vascular disease therapy. Inflammatory stress contributes to endothelial dysfunction in the pathogenesis of lung diseases [2,60]. The combination of proinflammatory cytokines, including interleukin 1 beta (IL-1β), tumor necrosis factor alpha (TNFα), and TGFβ, is a powerful EndMT inducer [28,29,30,32,34]. Good et al. found that the combination of IL-1β, TNFα, and TGFβ1 for six days induces EndMT in PAECs (I-EndMT) [30]. I-EndMT PAECs and lung fibroblasts isolated from patients with SSc-PAH showed elevated levels of cytokines, such as IL-6, IL-8, IL-13, and TNFα [30]. In addition, a cocktail of IL-1β, TNFα, and TGFβ1 induces CD44v and EndMT in PAECs [32]. CD44v-positive EndMT-induced PAECs showed upregulations of proinflammatory cytokines and chemokines, such as TNFα, IL-1β, IL-6, and CXCL12 [32]. The combination of TGFβ1 and IL-1β induces EndMT through Smad2/3 and ERK1/2 phosphorylation, which means that both Smad and non-Smad signaling are involved in this process [28]. Moreover, it has been demonstrated that cytokine levels, such as TGFβ1, IL-1β, IL-6, and TNFα, are increased in the lung tissue of MCT-induced PH rats [28,29]. Therefore, inflammatory cytokines induce EndMT and also induce cells to exhibit proinflammatory characteristics.

Among the various signaling pathways involved in EndMT, TGFβ signaling is known to be a major regulator of EndMT [61]. TGFβ upregulates EndMT-associated transcription factors, such as Snail, Slug, and Twist1, which leads to the upregulation of mesenchymal markers [2,61]. Although TGFβ induces EndMT mainly through the Smad-dependent canonical signaling pathway, Smad-independent non-canonical TGFβ signaling is also involved [61]. Non-canonical TGFβ signaling includes phosphatidylinositol 3-kinase (PI3K), mitogen-activated protein kinase (MAPK), and extracellular signal-regulated kinase (ERK) [62]. One group reported that *ATG7* knockdown promotes EndMT in PAECs [58]. During this process, mRNA and protein levels of TGFβ1 and its receptors, TGFβR1 and TGFβR2, are increased, and this elevates the phosphorylation of Smad2/3, leading to the upregulation of Slug and pro-fibrotic genes, connective tissue growth factor, and Collagen I [58]. Sabbineni et al. reported that the endothelial loss of *Akt1* increased TGFβ2 expression, which in turn elevated the phosphorylation of p38-MAPK and Smad2/3, resulting in EndMT [53]. These results indicate that both canonical and non-canonical signaling pathways are involved in TGFβ2-induced EndMT [53]. In addition, the inhibitor of beta catenin (β-catenin) suppressed the expression of mesenchymal markers and ameliorated vascular thickening in a SuHx PH model, suggesting that Akt1-mediated β-catenin signaling is a novel pathway for inducing EndMT [53]. Caveolin-1 plays an important role in the internalization of the TGFβ receptor [63]. The expression levels of Snail, Slug, SMAα and Collagen I were higher in pulmonary ECs isolated from *Caveolin-1* knockout mice than in WT mice [63]. Moreover, TGFβ1 treatment further increased SMAα and Collagen I expression in *Caveolin-1* knockout cells [63]. The phosphorylated Twist1 and vimentin were elevated in the lungs of PAH patients and MCT-induced PH rats, and TGFβ treatment increases Twist1 expression [7,64]. Mammoto et al. reported that the overexpression of Twist1 induces EndMT through TGFβR2-Smad2 signaling, and the phosphorylation of Twist1 Ser42 is required during hypoxia-induced EndMT [65].

BMPR2 is a member of the TGF receptor superfamily and is highly expressed on the pulmonary vascular endothelium [36,66,67,68]. BMPR2 mutations and low expression levels are closely associated with PAH [36,66,67,68,69]. Roughly 70–80% of familial PAH and 10–20% of sporadic cases of IPAH patients have BMPR2 mutations [36,66]. Several studies have demonstrated that BMPR2 expression is associated with EndMT in the lung vasculature of PAH animal models and patients [7,23,50,54]. Dysfunction of BMPR2 signaling induces EndMT through high mobility group AT-hook 1 (HMGA1) upregulation [23]. The knockdown of *HMGA1* or *Slug* prevented *BMPR2* silencing-induced SMAα expression [23]. In addition, pulmonary ECs isolated from endothelial-specific *BMPR2* knockout mice also showed EndMT with elevated HMGA1 and its target, Slug, expression [23]. *BMPR2*-deficient (BMPR2Δ140Ex1/+) rats exhibit spontaneous pulmonary vascular remodeling [7]. A recent study reported that *BMPR2* knockdown leads to the switch of cell junction protein from VE-cadherin to N-cadherin and increases Slug and Twist [11]. During this process, the heteromerization of BMP and TGFβ receptors was facilitated, leading to increased lateral TGFβ signaling responses [11]. Reynolds et al. reported that adenoviral BMPR2 delivery attenuates vascular remodeling in PAH animal models and treatment with BMPR2 ligands ameliorates TGFβ1-induced EndMT in vitro [36]. Accumulating evidence indicates that altered BMPR2 signaling is closely related to EndMT, and restoration of BMPR2 signaling can be a strategy for inhibiting EndMT.

Hypoxia contributes to EndMT in pulmonary ECs [26,27,70]. The hypoxia-inducible factor (HIF) family consists of HIF-1, HIF-2, and HIF-3, which are key regulators in maintaining oxygen homeostasis [50,71]. It has been reported that PVECs from PAH patients show EndMT with higher HIF-2α levels compared to control [50]. Although HIF-2α is degraded by prolyl hydroxylase domain protein 2 (PHD2) under normoxia, PHD2 was decreased in PVECs from IPAH, leading to the upregulation of Snail and Slug [50,71]. This suggests that PHD2 and HIF-2α are closely associated with EndMT. *PHD2* endothelial-specific knockout mice showed a severe PH phenotype, even under normoxia, while endothelial-specific *HIF-2α* knockout mice prevented developing hypoxia-induced PH [50]. Hypoxia upregulates HIF-1α, which acts as an upstream regulator of Twist1 by binding to its promoter and leads to EndMT [27]. Choi et al. described that HIF-1α is elevated in EndMT-derived cells in the lung tissue of radiation-induced fibrosis mice and human RIPF patients [41]. In addition, this study demonstrated that HIF-1α mediates TGFβ receptor/Smad signaling in radiation-induced EndMT [41]. These studies reflect the critical role of the HIF family in hypoxia-induced EndMT.

Notch is a family of transmembrane receptors and consists of Notch 1, 2, 3, and 4 [72,73]. Notch is activated by ligands, Jagged 1, Jagged 2, and Delta-like 1, 3, and 4, and produces the intracellular domain of Notch by proteolytic processing [72,73]. Notch signaling pathways have been associated with epithelial-to-mesenchymal transition and EndMT [72,73,74]. Noseda et al. demonstrated that activated Notch (Notch4IC, Notch1IC) and Jagged 1 lead to EndMT in human microvascular ECs [72]. Zhang et al. identified that Galectin-3 (Gal-3) is increased in the lung vasculature of patients with PAH and in the experimental animal model [33]. In vitro, Gal-3 treatment activated Jagged 1/Notch1 pathway, leading to EndMT [33]. The activated Jagged 1/Notch1 pathway was also identified in the PMVECs of bleomycin-induced PF rats [73]. *Jagged1* knockdown resulted in the downregulation of SMAα and NF-κB expression in bleomycin-treated rat PMVECs [73]. In addition, expressions of SMAα and Jagged 1/Notch1 were positively correlated [73].

NF-κB signaling is known to play a critical role in EndMT [51]. NF-κB transcriptionally regulates Snail, which is a transcription factor for promoting EndMT [51]. Several groups have shown the activation of NF-κB-Snail signaling in TGFβ1-induced ECs and MCT-rat models [51,75]. In addition, the NOD1 agonist, g-dglutamyl-meso-diaminopimelic acid (iE-DAP), induces EndMT via Akt/NF-κB signaling [76]. Taken together, Jagged 1/Notch signaling and NF-κB signaling are vital during EndMT.

MicroRNAs (miRNAs) are 22-nucleotide, small, non-coding RNAs and important regulators of EndMT in many diseases [77,78]. It has been demonstrated that miRNAs, such as miR-21, miR-27a, miR-126a-5p, miR-130a, miR-139-5p, and miR-199a-5p, are involved in EndMT in vitro and in vivo. Parikh et al. used a network-based bioinformatic method to identify PH-modifying miRNAs and found that miR-21 is upregulated in the pulmonary vessels of PH animal models and human PAH patients [79]. In PAECs, hypoxia, inflammation, and BMPR2-dependent signaling induced miR-21 and suppressed its target, RhoB [79]. Another group provided evidence that miR-21 levels, Akt phosphorylation/activation, Snail expression, and NF-κB signaling were elevated in TGFβ1-induced EndMT [75]. Our group determined that iE-DAP downregulates miR-139-5p and activates Akt/NF-κB signaling, which leads to EndMT [76]. In addition, the overexpression of miR-139-5p reversed the nuclear translocation of NF-κB, resulting in the inhibition of iE-DAP-induced EndMT [76]. Li et al. reported that the mouse lung tissue of MCT-induced PAH showed increased miR-130a expression, and its regulation was NF-κB-dependent [80]. Moreover, the overexpression of miR-130a induced EndMT in PMVECs [80]. Further, a lung tissue microarray identified that miR-126a-5p is upregulated in a neonatal PH rat model [81]. Hypoxia induced the expression of miR-126a-5p and led to EndMT through PI3K/Akt signaling in primary cultured rat PMVECs [81]. Furthermore, circulating miR-126a-5p levels were increased in the sera of PAH patients [81]. Several studies have reported that miR-27a is increased in pulmonary arteries of PAH. [26,82]. Moreover, there is upregulation of miR-27a in pulmonary arteries of PAH rats and hypoxia-induced PAECs [26]. MiR-27a acts as an EndMT inducer through the suppression of Smad5 and the upregulation of Snail and Twist [26]. In addition, the contribution of Snail-induced miR-199a-5p to radiation-induced EndMT has been evaluated previously [83].

**Figure 2:**
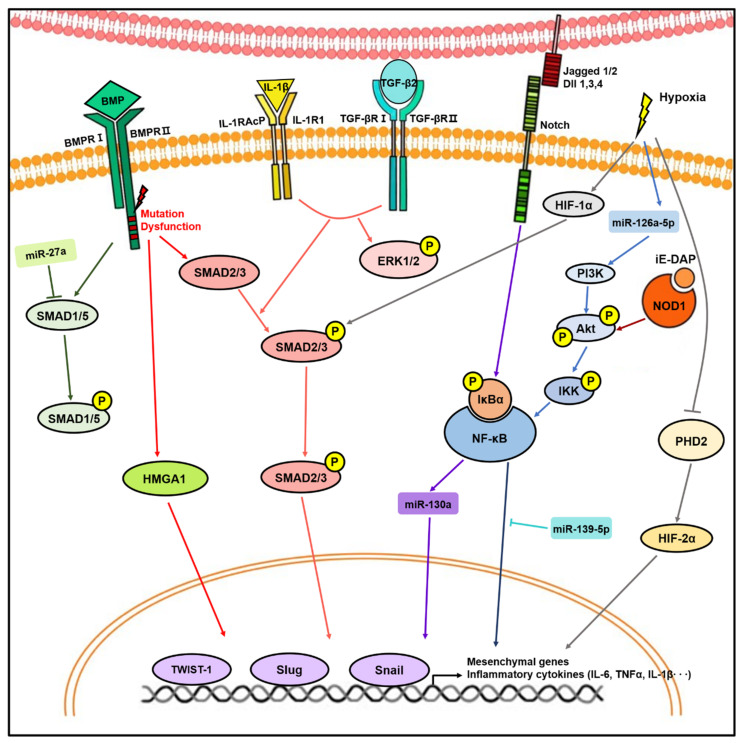
Molecular signaling pathways involved in endothelial-to-mesenchymal transition (EndMT). Stimulation with transforming growth factor-β (TGFβ), bone morphogenic protein (BMP), Notch ligands, inflammatory stress, and hypoxia induce expression of transcription factors, such as Twist1, Slug, and Snail, resulting in EndMT. During this process, mediators including microRNAs (miRNAs), Smad, Akt/nuclear factor kappa B (NF-κB), and hypoxia-inducible factor (HIF) play important roles in EndMT.

In conclusion, many studies have demonstrated the interplay of various signaling pathways in the process of EndMT (Figure 2). However, better knowledge of how they engage in crosstalk with one another and what other mediators are involved is required for developing therapeutic strategies.

## 5. Targeting EndMT for Potential Therapeutic Applications

Many studies have reported potential therapeutic strategies to alleviate PF and PAH by targeting EndMT [7,33,34,41,52]. Adenoviral BMPR2 administration inhibited vascular remodeling and improved cardiac function in chronic hypoxia or MCT-induced PH rats [36]. In addition, recombinant BMP2 or BMP7 treatment reversed TGFβ1-induced EndMT in PMVECs [36]. One study suggested that modulating miRNAs may be a therapeutic strategy for PAH. When the MCT-exposed rats were infected with lentivirus-overexpressing miR-181b, RVSP, mean PAP, the decrease in pulmonary arterial wall thickness and the overexpression of miR-181b inhibited EndMT in rat PAECs by negatively regulating endocan and TGFβR1 [29]. Our group demonstrated that ginsenoside Rg3 attenuates iE-DAP-induced EndMT by upregulating miR-139-5p [76]. Nintedanib, a tyrosine kinase inhibitor, significantly decreased the phosphorylation of platelet-derived growth factor and fibroblast growth factor receptors, which were increased in the pulmonary artery of PAH, resulting in the improvement of PAH in vivo [34]. In addition, nintedanib downregulated the expression of mesenchymal markers in PMVECs [34]. One study suggested that hydrogen sulfide inhibits the NF-κB-Snail pathway and EndMT, resulting in a therapeutic effect on PAH [51]. In addition, treatment with the prostaglandin E2 receptor 4 (EP4) agonist decreases EndMT in vitro and reduces right ventricular fibrosis in a rat model of PAH [64]. The expression of Gal-3 was increased in PAH patients and a hypoxia-induced PAH rat model, and treatment with Gal-3 inhibitor, N-Lac, recovered RVSP, pulmonary artery acceleration, and pulmonary arterial velocity time integral [33]. Salvianolic acid A restores pulmonary vascular remodeling and improves vascular relaxation by upregulating Nrf2/HO-1 signaling while reducing TGFβ1 and EndMT in MCT-induced PAH rat models [35]. CD44v binds to and stabilizes the cystine transporter subunit (x-CT) when PAECs undergo EndMT. Sulfasalazine inhibits x-CT and restores EndMT in vitro and in vivo [32]. Rapamycin increased the expression of p120-catenin, a cytoplasmic scaffold protein that regulates cell-to-cell adhesion by binding VE-cadherin, and led to the downregulation of Twist1 expression in the lungs of MCT-induced PH rats [7]. Moreover, rapamycin inhibited the migration and proliferation of PAECs isolated from normal and PAH patients [7]. As such, rapamycin has curative effects on EndMT in MCT-induced PH rat models [7].

CD26/dipeptidyl peptidase 4 (DPP-4) is widely distributed in various cell types of the lung and promotes TGFβ signaling and EndMT [44,84]. Vildagliptin, a DPP-4 inhibitor, inhibits LPS-induced EndMT in PMVECs and attenuates the LPS-induced PF mouse model [44]. Another study confirmed that treatment with a DPP-4 inhibitor, sitagliptin, reduces pulmonary arterial remodeling and alleviates EndMT in PH-induced rats [52]. In addition, treatment with atazanavir sulphate, an antiretroviral protease inhibitor, ameliorates EndMT in cobalt chloride (CoCl_2_)-induced hypoxic PMVECs and decreases fibrotic lesions in a bleomycin-induced rat PF model, suggesting a potential therapeutic effect of atazanavir sulphate on EndMT [43]. Further, the overexpression of HSPB1, which protects against cellular stress, decreased radiation-induced EndMT in vitro and in vivo [45]. The HIF-1α inhibitor reduced the expression of HIF-1α, the phosphorylation of Smad2/3, and EndMT in radiation-induced PAECs; furthermore, it decreased the SMAα+ CD31+ double-positive cells and fibrosis area in the lungs of the RIPF mouse model [41].

**Figure 3:**
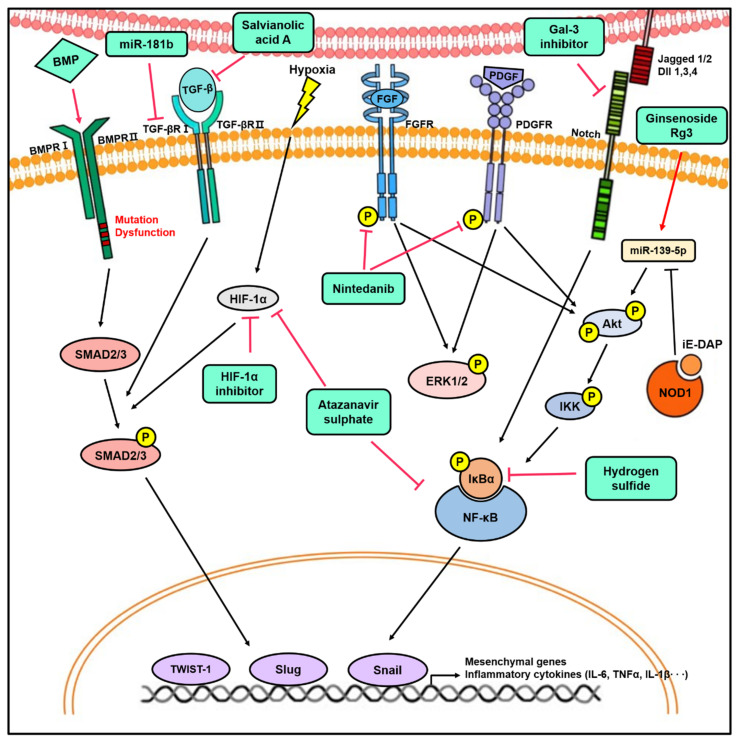
The novel therapeutic approach for lung diseases by targeting EndMT. The modulation of signaling pathways involved in EndMT by miRNAs, inhibitors, and agonists has therapeutic effects in vitro and in vivo. Targeting EndMT reduces mesenchymal marker expression and pulmonary vascular remodeling, which ultimately ameliorates the hemodynamic phenotypes in the animal model of PH. In addition, the inhibition of EndMT decreases fibrotic lesion in various PF animal models. This provides insight into the therapeutic potential of targeting EndMT.

Taken together, EndMT plays a critical part in vascular diseases, especially PF and PAH, and many studies on various therapeutic strategies targeting EndMT have been conducted by modulating various signaling pathways and epigenetic factors (Figure 3). However, further studies are still necessary to clarify the mechanism between EndMT and pulmonary vascular diseases to find more effective therapeutic agents that can treat vascular diseases, such as PF or PAH, by targeting EndMT. Table 1 and Table 2 each list a summary of studies on EndMT in lung diseases and targeting EndMT as a therapeutic strategy.

## 6. Conclusions and Perspective

Here, we review the role of EndMT and its downstream pathways, and the therapeutic implications of targeting EndMT for lung diseases. As described earlier, many studies have demonstrated that EndMT is associated with the pathogenesis of PAH and PF in both in vitro and in vivo models, as well as in the lung tissue of humans. Thus, targeting EndMT can be a new therapeutic approach to treating lung diseases given the fact that there are no drugs to cure PAH or PF. Indeed, emerging evidence suggests that pharmacological approaches to inhibiting EndMT have the potential to treat lung diseases in vitro and in vivo. EndMT can also contribute to other human diseases, such as cancer, atherosclerosis, neointima formation, vascular calcification, and cerebral cavernous malformations. Therefore, inhibiting EndMT might represent a broadly applicable therapeutic strategy for the treatment of not only pulmonary vascular diseases, but also many diseases associated with endothelial dysfunction. Although it is now clear that EndMT is closely associated with the pathogenesis of multiple diseases, the EndMT-targeting therapeutic approach needs to be carefully evaluated before clinical application. Given that ECs display organ-specific heterogeneity in function and phenotype, in health and disease, and in their response to environmental stimuli, it is possible that certain vessel types and vascular beds are more sensitive to EndMT-inducing signals. Therefore, it is important to understand the exact molecular mechanisms related to the EndMT process in the context of the heterogeneity of ECs. Importantly, EndMT may be a reversible biological process, what is called mesenchymal-to-endothelial transition, suggesting that exploration of the regulatory mechanism of the reversible process of EndMT will provide new insights into the prevention and treatment of various human diseases, and may be applied to tissue engineering. In conclusion, we believe that studies of EndMT in the context of endothelial heterogeneity will provide us with better insights into the molecular mechanisms of a broad variety of human diseases, and will help to develop novel vascular bed-specific therapies.

## Figures and Tables

**Table 1:** Summary of the main studies on EndMT in lung diseases.

Lung Diseases	In Vivo Model	In Vitro Model	Cell Line	Endothelial Markers	Mesenchymal Markers	EndMT Mediators	Reference
PH	SuHx, MCT rats	BMPR2 deficiency	PAECs	CD31, VE-cad, CD34, Tie2	SMAα, Vimentin, p-Vimentin	Twist1	[7]
PH	BMPR2 KO mice	BMPR2 deficiency	EAhy926	VE-cad	N-cad	Slug, Twist	[11]
PH	EC-specific BMPR2 KO mice	BMPR2 deficiency	PAECs	vWF, CD31, VE-cad	SM22α, SMAα, p-Vimentin	HMGA1, Snail, Slug	[23]
PH	Hypoxia-exposed rats	Hypoxia	Rat PMVECs	CD31	SMAα, Collagen I, III	HIF-1α, Twist1	[27]
PH	SuHx mice	Combination of TGFβ1, TNFα, and IL-1β	PAECs	vWF, CD31, VE-cad, Occudin	SMAα, Calponin, Collagen I	Inflammatory cytokines	[30]
PH	VE-cad Cre or Tie2 Cre-mTomato/mGFP lineage tracing mice			vWF, CD31, VE-cad, Tie2	SMAα	Not determined	[48]
PH	MCT rats, EC-specific phd2, egln1, KO mice	Hypoxia, TGFβ1	PVECs	CD31, VE-cad	SM22α, Vimentin, FN, SMAα, FSP1	HIF-2α, Snail, Slug, PHD2	[50]
PF	Radiation-exposed mice	Radiation	PAECs, PMVECs	CD31, VE-cad	SMAα, Vimentin, FSP1, Collagen, MMP9	TGFβ-RI, Smad2/3, HIF-1α, Snail	[41]
PF	Bleomycin-treated Tie2-Cre/CAG-CAT-Lac mice	Combination of Ras and TGFβ	MS-1	CD31, VE-cad, CD34, Tie2,	SMAα, FN, Collagen I	Not determined	[42]
PF	Radiation-exposed mice	HSPB1 deficiency	PAECs, PMVECs	CD31, VE-cad	SMAα	Inflammatory cytokines	[45]

PH, pulmonary hypertension; PF, pulmonary fibrosis; SuHx, SU-5416/hypoxia model; MCT, monocrotaline; BMPR2, bone morphogenic protein receptor-2; KO, knockout; EC, endothelial cell; TGFβ, transforming growth factor beta; TNFα, tumor necrosis factor alpha; IL-1β, interleukin-1 beta; PHD2, prolyl hydroxylase domain protein 2; HSPB1, endothelial heat shock protein beta 1; PAECs, pulmonary arterial endothelial cells; PVECs, pulmonary vascular endothelial cells; PMVECs, pulmonary microvascular endothelial cells; MS-1, mouse microvascular endothelial cell line; CD31, cluster of differentiation 31; CD31, cluster of differentiation 34; VE-cad, vascular endothelial cadherin; vWF, von willebrand factor; SMAα, α-smooth muscle actin; N-cad, neural cadherin; SM22α, smooth muscle protein 22-α; FN, fibronectin; FSP1, fibroblast-specific protein 1; MMP, matrix metallopeptidase 9; HIF, hypoxia-inducible factor; HMGA1, high-mobility group AT-hook 1; p-Vimentin, phosphorylated vimentin.

**Table 2:** Key studies targeting EndMT as a therapeutic strategy in PH and PF.

Clinical Relevance	In Vitro Model	In Vivo Model	Negative Regulator of EndMT	Reference
PH		MCT rat	Rapamycin	[7]
PH	Combination of TGFβ1, TNFα, and IL-1β-treated rat PAECs	MCT rat	miR-181b	[29]
PH	Combination of TGFβ1, TNFα, and IL-1β-treated PAECs	SuHx mice	Sulfasalazine	[32]
PH		Hypoxia-exposed rat	Galectin-3 inhibitor	[33]
PH	Combination of TGFβ2, TNFα and IL-1β-treated PMVECs	SuHx rat	Nintedanib	[34]
PH	TGFβ1-treated PAECs	MCT rat	Salvianolic acid A	[35]
PH	TGFβ1-treated PAECs	MCT rat	Hydrogen sulfide	[51]
PH		MCT rat	Sitagliptin	[52]
PH	TGFβ-treated HUVECs	MCT rat	EP4 agonist	[64]
PH	TGFβ1-treated PMVECs	MCT rat, Hypoxia-exposed rat	BMPR2, rhBMP2, rhBMP7	[36]
PF	Radiation-exposed PAECs	Radiation-exposed mice	HIF-1α inhibitor	[41]
PF	CoCl_2_-treated PMVECs	Bleomycin-treated rat	Atazanavir sulphate	[43]
PF	LPS-treated PMVECs	LPS-treated mice	Vildagliptin	[44]
PF	Radiation-exposed PMVECs	Radiation-exposed EC conditionally overexpressed HSPB1 mice	HSPB1	[45]

PH, pulmonary hypertension; PF, pulmonary fibrosis; SuHx, SU-5416/hypoxia model; MCT, monocrotaline; EC, endothelial cell; LPS, lipopolysaccharides; CoCl_2_, cobalt chloride; TGFβ, transforming growth factor beta; TNFα, tumor necrosis factor alpha; IL-1β, interleukin 1beta; HSPB1, heat shock protein beta 1; HIF1α, hypoxia-inducible factor 1 alpha; BMPR2, bone morphogenic protein receptor-2; rhBMP, recombinant human bone morphogenic protein; EP4, prostaglandin E2 receptor 4; PAECs, pulmonary arterial endothelial cells; PMVECs, pulmonary microvascular endothelial cells; HUVECs, human umbilical vein endothelial cells.
